# Chitosan, Methyl Jasmonate, and Silicon Induce Resistance to Angular Leaf Spot in Common Bean, Caused by *Pseudocercospora griseola*, with Expression of Defense-Related Genes and Enzyme Activities

**DOI:** 10.3390/plants13202915

**Published:** 2024-10-18

**Authors:** Gülsüm Palacıoğlu

**Affiliations:** Department of Plant Protection, Fethiye Faculty of Agriculture, Muğla Sıtkı Koçman University, 48300 Muğla, Türkiye; gulsumpalacioglu@mu.edu.tr

**Keywords:** *Phaseolus vulgaris* L., biotic stress, defense genes, elicitor, gene expression, induced resistance

## Abstract

This study assessed the efficacy of chitosan, methyl jasmonate, and silicon in the reduction of disease severity and the induction of defense responses in common bean plants against angular leaf spot caused by *Pseudocercospora griseola.* The expression level of several pathogenesis-related (PR) proteins, PR1, PR2 (β-1,3-glucanase), and PR3 (chitinase), and defense-related enzymes, phenylalanine ammonia-lyase, peroxidase, and lipoxygenase, was analyzed at different time points in common bean plants after different treatments. Elicitor treatments significantly reduced disease severity 21 days after inoculation, with silicon at a 2 mM concentration proving most effective with 38.93% disease control, followed by 1 mM MeJA and 2% chitosan, respectively. Treatments with chitosan, methyl jasmonate, and silicon, regardless of pathogen infection, significantly elevated *PR1*, *PR2*, and *PR3* gene expressions at 48 h after inoculation (hpi). PAL and POD activities were similarly increased following elicitor treatments and pathogen infection, especially at 48 hpi. Chemical elicitors applied post-inoculation induced PR proteins, PAL, and POD enzyme activities at 48 hpi, while LOX activity exhibited a variable fluctuation with treatments. These findings suggested that chemical elicitors, especially silicon, were effective in reducing ALS disease severity in common beans, with improved resistance associated with the expression of pathogen-responsive genes. This study is the first to analyze the expression profiles of defense-related genes in common beans treated with chemical elicitors prior to *P*. *griseola* infection.

## 1. Introduction

The common bean (*Phaseolus vulgaris* L.) is considered one of the most important grain legumes [1]. Among fungal diseases, restricting common bean production, angular leaf spot (ALS) caused by the pathogen *Pseudocercospora griseola* (Sacc.) Crous & U. Braun 2006 (syn: *Phaeoisariopsis griseola*) is one of the most devastating diseases, leading to yield losses of up to 80% under favorable conditions [2,3,4,5]. To manage angular leaf spot, various control strategies such as fungicide application, disease-free seed use, crop rotation, host-plant resistance, and cultivar mixtures have been implemented [5,6,7]. Genetic resistance, though effective and economical, is frequently undermined by the emergence of various pathogen races that could overcome host resistance and the lack of sufficient resistance sources [7,8,9,10,11,12]. Consequently, substantial efforts have been directed towards discovering new alternatives to mitigate the adverse effects of chemicals globally. Elicitors, offering an eco-friendly alternative to traditional pesticides, significantly enhance plant resistance against diseases by activating various defense mechanisms. Comprehensive studies have been conducted on the potential practical use of elicitor treatments in plants as an alternative disease management strategy against pathogens, and several natural compounds have been proven to be effective in inducing resistance to different plant pathogens [13].

Chitosan, β-(1,4)-2-amino-2-deoxy-D-glucose, is a natural compound exhibiting antimicrobial and antioxidant activities against plant pathogens [14,15]. Chitosan and its derivatives reduce pathogen growth and contribute to plant defense systems [14]. Chitosan reduces disease incidence and symptom progression by impeding spore germination, germ tube elongation, and mycelial growth of fungi [16,17,18,19]. Chitosan contributes to the regulation of pathogenesis-related proteins (PRs) such as β-1,3-glucanase and chitinase, defense-related enzymes like phenylalanine ammonia-lyase (PAL), peroxidase (POD), polyphenol oxidase (PPO), catalase (CAT), and superoxide dismutase (SOD), and the accumulation of secondary metabolites including phytoalexins, lignin, suberization, callose, and phenolic compounds [14,19,20]. Chitosan applications have demonstrated efficacy in inducing resistance to a variety of plant pathogens, including *Erysiphe cichoracearum*, *Colletotrichum gloeosporioides*, *Botrytis cinerea*, *Fusarium andiyazi*, *Pyricularia oryzae*, and *Rhizoctonia solani* [21,22,23,24,25].

Jasmonic acid (JA) is a crucial signal molecule that regulates many physiological processes during plant development and enhances plant resistance against various biotic and abiotic factors [26,27,28]. The roles of jasmonic acid and its methyl ester, methyl jasmonate (MeJA), are well documented in plant resistance against pathogens, primarily coordinating the expression of defense-related genes associated with the induction of systemic acquired resistance (SAR) [13,29,30]. Methyl jasmonate induces genes encoding PR proteins such as chitinases and β-1,3-glucanases and regulates the production of various secondary metabolites including PAL, LOX, chalcone synthase, POD, and PPO [31,32,33,34,35]. Treatment of plants with MeJA has been known to confer broad resistance against fungal pathogens in a wide range of plant species such as oilseed rape [36], tomato [37], *Medicago truncatula* [38], and wheat [35].

Silicon (Si) is not an essential element for plants; however, it significantly contributes to plant growth and resistance against biotic and abiotic stress by involving metabolic and physiological processes and regulating the complex network of signal transduction pathways [39,40,41,42]. Silicon enhances the mechanical structure by forming a hard outer layer in addition to stimulating antimicrobial compounds against pathogen infection [41,42,43,44]. Numerous studies have reported improved plant resistance to fungal diseases such as *Podosphaera xanthii*, *Bipolaris oryzae*, *Pyricularia oryzae*, *Fusarium oxysporum* f. sp. *vasinfectum*, and *Sclerotinia sclerotiorum* through silicon applications [45,46,47,48,49]. One study on common beans indicated that foliar application of potassium silicate after pathogen infection reduced the severity of angular leaf spot disease [50]. However, the effectiveness of silicon on pathogens is dependent significantly on several factors including silicon form, concentration, application methods, pH, and environmental conditions. Silicon application to the roots has been found to be more effective than foliar applications in managing plant diseases [51,52,53].

This study explored the induction of disease resistance in common beans against *Pseudocercospora griseola* through treatments with chitosan, methyl jasmonate, and silicon at different concentrations. To elucidate the molecular underpinnings of this resistance, I analyzed the expression of particular resistance genes at different time points by quantitative real-time PCR. The findings offered comprehensive insights into the function of defense genes and enzymes linked to resistance against ALS in common beans.

## 2. Materials and Methods

### 2.1. Plant Growth

A common bean cultivar, Gina, was selected for its known susceptibility to angular leaf spot in the experiment [54]. Seeds were surface-sterilized with 1% NaClO for 2 min, washed with distilled water, and germinated in moist chambers. Following germination, seedlings were transferred into 15 cm pots filled with sterilized compost for chitosan and MeJA applications. To assess the effect of silicon treatment on disease development, germinated seedlings were grown hydroponically in a 10 L tank containing nutrient solution (3 mmol L^−1^ KNO_3_, 2 mmol L^−1^ Ca(NO_3_)_2_.4H_2_O, 0.5 mmol L^−1^ MgSO_4_.7H_2_O, 0.25 mmol L^−1^ (NH_4_)_2_SO_4_, 0.5 mmol L^−1^ NH_4_H_2_PO_4_, 12.5 μmol L^−1^ H_3_BO_3_, 1 μmol L^−1^ ZnSO_4_.7H_2_O, 0.25 μmol L^−1^ CuSO_4_.5H_2_O, 0.25 μmol L^−1^ MnCl_2_.4H_2_O, 0.25 μmol L^−1^ (NH_4_)_6_Mo_7_O_24_.4H_2_O, 25 μmol L^−1^ FeSO_4_.7H_2_O, and 25 μmol L^−1^ EDTA bisodium) without Si [55]. After three days, seedlings were transferred to a tank containing a nutrient solution with or without Si under aeration. The plants were grown in a growth room at 20 ± 1 °C with a 14 h photoperiod up to the V3 growth stage for pathogen inoculation.

### 2.2. Pathogen Inoculation

*Pseodocercospora griseola*, isolate Pg-5, was obtained from the culture collection of the Department of Plant Pathology at the Agricultural Faculty, Ankara University. To prepare the inoculum, the pathogen was cultivated on bean agar medium at 23 °C for 15 days. Inoculum concentration was adjusted to a final concentration of 2 × 10^4^ spores/mL by diluting with sterile distilled water. Seedlings at the V3 growth stage were sprayed with the pathogen inoculum until run-off occurred and then covered with polyester bags to maintain humidity for 24 h. After inoculation, the first trifoliate leaves of plants were collected at 12, 24, 48, and 72 h. Three pods were used for each time point and leaves from 15 plants per replicate were pooled. The samples were immediately frozen in liquid nitrogen and stored until used for RNA isolation. Mock inoculations were performed using only distilled water. Disease severity was assessed 21 days after inoculation using a 1–9 scale as described by [56]. The data obtained were subjected to analysis of variance and evaluated by the least significant difference test (*p* < 0.05) with the Minitab 17 (Minitab Inc., State College, PA, USA) package program.

### 2.3. Treatment with Chemical Elicitors

Common bean seedlings were treated with chitosan, MeJA, and silicon 2 days prior to pathogen inoculation. Chitosan (low molecular weight, 75–85% deacetylation; HiMedia, Kennett Square, PA, USA) was prepared as a 10 mg/mL stock solution by diluting it with an equal amount of acetic acid. The mixture was stirred for 24 h at room temperature and adjusted to pH 5.6 with 1 N NaOH [18]. The chitosan solution was then diluted at concentrations of 1, 1.5, and 2 mg/mL, and the seedlings were treated with a hand sprayer until incipient run-off [18,21,22]. MeJA (Sigma Chemical Co., St. Louis, MO, USA) was prepared as a 1 M stock solution [32] and diluted to concentrations of 0.1, 1, and 10 mM [34,37,38], which were then applied to seedlings as described above. Silicon was added to the nutrient solution as soluble potassium silicate (26% SiO_2_, 13% K_2_O a.i; Eastroot, Antalya, Türkiye) at a concentration of 2 mM [45,50,55]. For silicon application, seedlings were grown in a hydroponic system containing a nutrition solution without Si for 3 days to allow for adaptation. Following this period, seedlings were transferred to a tank containing nutrient solution with or without Si under aeration. The nutrient solution was changed every week, and the pH was maintained at approximately 5.6. Common bean plants were inoculated with the pathogen 2 days after chemical elicitor treatments.

### 2.4. Total RNA Extraction and cDNA Synthesis

Total RNA was extracted from leaf samples collected at different time points using NucleoZOL reagent (Macherey-Nagel, Düren, Germany) following the manufacturer’s protocol. The RNA samples were treated with *DNase I* (Thermo Fisher Scientific Inc., Wilmington, DE, USA) according to the protocol described by the manufacturer. RNA quantity and quality were assessed using a DS-11 FX+ spectrophotometer (DeNovix Inc., Wilmington, DE, USA). Complementary DNA (cDNA) was synthesized from each RNA sample using an iScript™ cDNA synthesis kit (Bio-Rad Laboratories Inc., Hercules, CA, USA) according to the manufacturer’s guidelines. The cDNA samples were then diluted 10-fold to a final volume of 200 μL with sterile Milli-Q^®^ water (Millipore, Bedford, MA, USA).

### 2.5. Quantitative Real-Time PCR and Data Analysis

Quantitative PCR was performed using a LightCycler^®^ 96 Real-Time PCR thermal cycler (Roche Diagnostics, Maroussi, Greece). Each qPCR reaction contained 1× Sso Advanced SYBR Green Supermix (BioRad, Hercules, CA, USA), 2 μL of cDNA, and 0.5 μM of primer (Table 1) in a total reaction volume of 10 μL [57]. The thermal cycling profile was 95 °C for 3 min, then 95 °C for 10 s, and at 60 °C for 10 s repeated for 45 cycles, followed by a melting step in which the temperature was increased from 65 to 95 °C. Three biological and two technical replications were performed. Relative quantification of gene expression was calculated using the 2^−ΔΔCT^ method [58] using Ct-values of the *actin* gene for normalization. Fold changes were transformed by using a binary logarithm (log2) and analyzed with Student’s *t*-test using Minitab 17 statistical software (Minitab Inc., State College, PA, USA). Each value represents the average of two independent experiments, with standard deviations calculated from three replicates.

## 3. Results

### 3.1. Effect of Chemical Elicitors on Disease Development of Angular Leaf Spot

**Figure 1:**
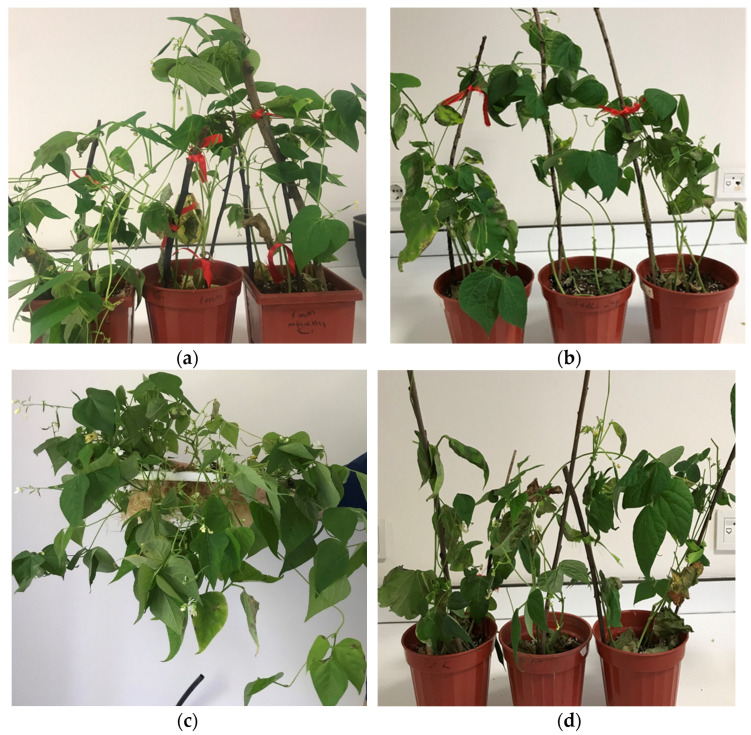
Disease symptoms caused by *Pseudocercospora griseola* on common bean plants treated with methyl jasmonate (**a**), chitosan (**b**), and silicon (**c**) at 1 mM, 2 mg/mL, and 2 mM concentrations, respectively. Control plants inoculated with the pathogen (**d**). Disease severity was assessed 21 days after the pathogen inoculation.

**Figure 2:**
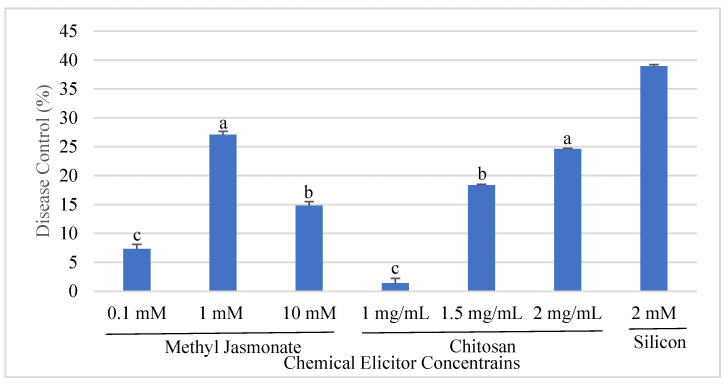
Disease control (%) on common bean plants in response to methyl jasmonate (MeJA), chitosan, and silicon treatment against *Pseodocercospora griseola*. Disease severity was assessed 21 days after the pathogen inoculation. Data represent the mean ± SD of three replicates. Different letters among elicitor concentrations showed statistically significant differences (Student’s *t*-test, *p* < 0.05).

Disease development of angular leaf spot was notably affected by different concentrations of elicitors. Brown lesions were visible on the inoculated leaves within 9–10 days, whereas control plants exhibited no symptoms. After 21 days of the pathogen inoculation, the maximum disease control (38.3%) was achieved with a 2 mM silicon treatment, followed by 1 mM MeJA and 2 mg/mL chitosan treatments, which resulted in 27.1% and 24.62% disease control, respectively, compared to untreated plants (Figure 1 and Figure 2). Statistically significant differences (*p* < 0.05) were observed among the disease control levels provided by different concentrations of elicitors. Plants treated with chitosan and silicon exhibited less extended lesions, whereas increasing MeJA doses did not show significant differences compared to controls. The concentrations that offered the maximum disease control were selected for analyzing the expression level of defense genes associated with resistance induction in common beans.

### 3.2. Expression Analysis of Defense-Related Genes Associated with Elicitor Treatment and Pathogen Inoculation in Common Bean

**Figure 3:**
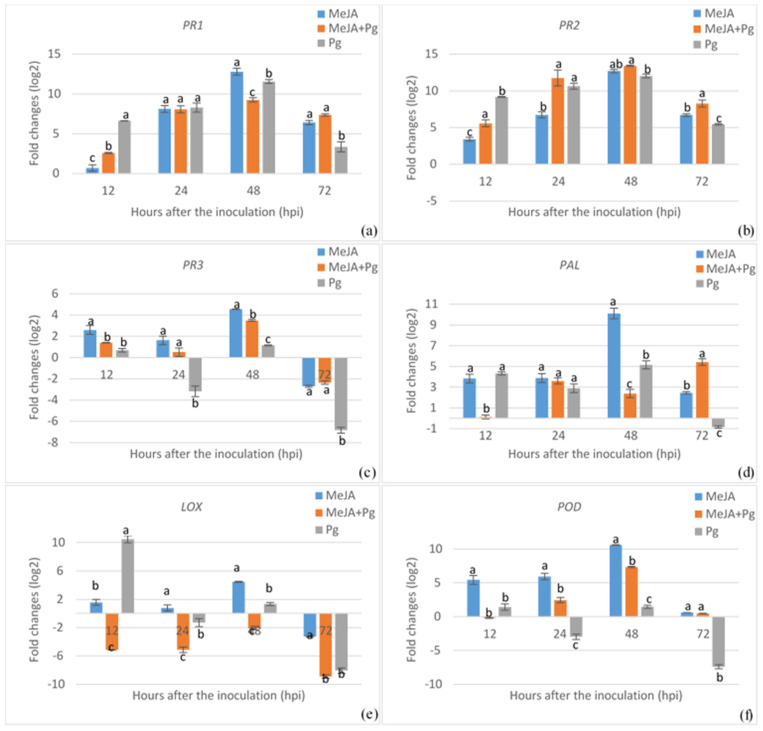
Expression profiles of *PR1* (**a**), *PR2* (**b**), *PR3* (**c**), *PAL* (**d**), *LOX* (**e**), and *POD* (**f**) genes in common bean plants treated with methyl jasmonate (MeJA) prior to *Pseodocercospora griseola* (Pg) inoculation. The transcript levels of defense-related genes were normalized to the expression of the actin gene. Data represent the mean ± SD of three replicates. Different letters on the bars show statistically significant differences (Student’s *t*-test, *p* < 0.05).

**Figure 4:**
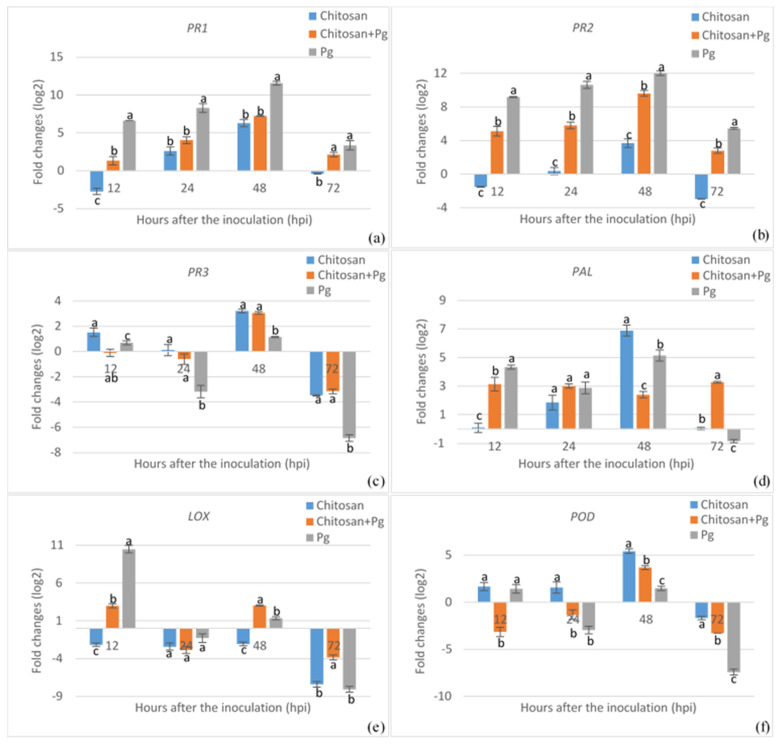
Expression profiles of *PR1* (**a**), *PR2* (**b**), *PR3* (**c**), *PAL* (**d**), *LOX* (**e**), and *POD* (**f**) genes in common bean plants treated with chitosan prior to *Pseodocercospora griseola* (Pg) inoculation. The transcript levels of defense-related genes were normalized to the expression of the actin gene. Data represent the mean ± SD of three replicates. Different letters on the bars show statistically significant differences (Student’s *t*-test, *p* < 0.05).

**Figure 5:**
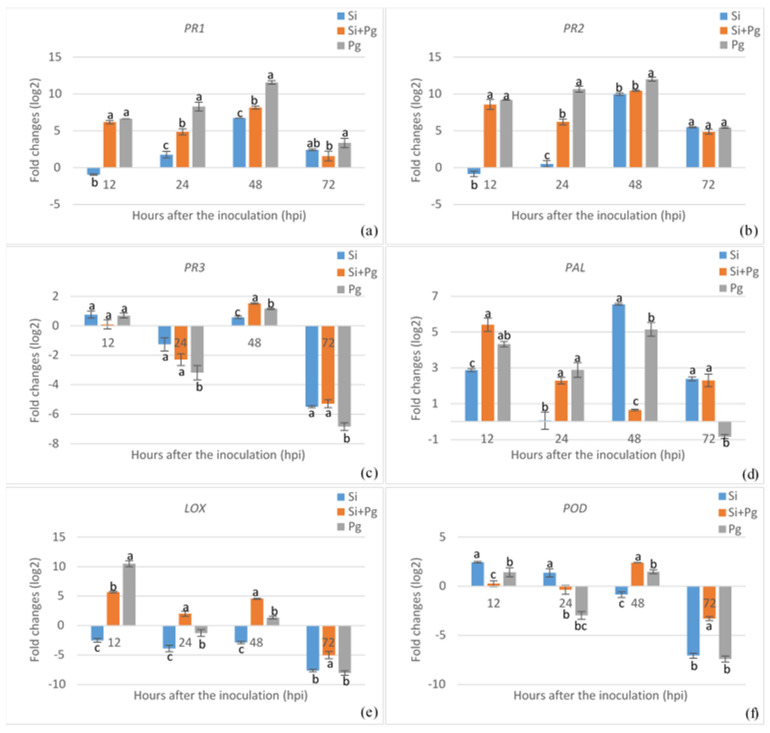
Expression profiles of *PR1* (**a**), *PR2* (**b**), *PR3* (**c**), *PAL* (**d**), *LOX* (**e**), and *POD* (**f**) genes in common bean plants treated with silicon (Si) prior to *Pseodocercospora griseola* (Pg) inoculation. The transcript levels of defense-related genes were normalized to the expression of the actin gene. Data represent the mean ± SD of three replicates. Different letters on the bars show statistically significant differences (Student’s *t*-test, *p* < 0.05).

To assess the expression patterns of various defense-related genes regulated in common beans either by chemical elicitors, pathogens, or both, we conducted a gene expression analysis using RT-qPCR, involving chitosan, MeJA, or silicon treatments, with or without *P*. *griseola* inoculation. The expression of the *PR1* gene increased markedly with nearly all treatments until 48 hpi, followed by a decrease at 72 hpi (Figure 3a, Figure 4a and Figure 5a). The highest *PR1* gene expression was recorded in MeJA-treated plants with a 12.78-fold enhancement, followed by silicon and chitosan treatments. In MeJA-pretreated plants, pathogen inoculation led to a 9.24-fold upregulation of the *PR1* gene at 48 hpi, while chitosan and silicon treatments prior to pathogen inoculation resulted in 8.15- and 7.23-fold increases in the *PR1* transcript level, respectively. *P*. *griseola* inoculation alone also induced *PR1* gene expression up to 48 hpi, followed by a reduction at 72 hpi. Similarly, the *PR2* gene exhibited a marked upregulation with MeJA treatment across all time points, while chitosan and silicon treatments led to either downregulation or slight induction in the *PR2* transcript until 48 hpi (Figure 3b, Figure 4b and Figure 5b). In plants pretreated with elicitors, the pathogen inoculation significantly increased the transcript level of the *PR2* gene at all the time points. The highest expression of the *PR2* gene was observed at 48 hpi in response to MeJA, chitosan, and silicon treatments, with enhancements of 13.4-fold, 9.62-fold, and 10.4-fold, respectively. Similarly, *P*. *griseola* inoculation alone resulted in a significant increase in *PR2* gene transcripts in the plants. The *PR3* gene exhibited no significant induction with any of the treatments until 48 hpi (Figure 3c, Figure 4c and Figure 5c). *PR3* gene expression was considerably higher in plants treated with MeJA compared to those treated with chitosan and silicon. The peak expression of the *PR3* gene reached 4.53-fold at 48 hpi with MeJA treatment. *PR3* expression showed a weak elevation or decrease in treatments of the elicitors with *P*. *griseola* and the pathogen alone. The transcript level of the *PR3* gene was gradually downregulated in all treatments by 72 hpi.

PAL activity peaked at 48 hpi for MeJA, chitosan, and silicon treatments, with fold increases of 10.11, 6.9, and 6.55, respectively (Figure 3d, Figure 4d and Figure 5d). However, early induction was observed in pathogen-inoculated plants. The maximum level with the pathogen inoculation in silicon-pretreated plants was reached at 12 hpi with an enhancement of 5.40-fold, and then gradually downregulated. Chitosan- and MeJA-pretreated plants exhibited a similar expression trend at all time points. *P*. *griseola* inoculation alone caused comparable fluctuations in PAL transcript levels across all time points. MeJA treatment led to a 4.46-fold upregulation of LOX activity at 48 hpi, while chitosan and silicon treatments caused a downregulation across all time points (Figure 3e, Figure 4e and Figure 5e). Similarly, pathogen application in silicon-pretreated plants resulted in a 5.68-fold increase in LOX transcript levels at 12 hpi, while chitosan-pretreated plants showed increases of 2.99- and 3.03-fold at 12 and 48 hpi, respectively. In MeJA-pretreated plants, pathogen inoculation dramatically downregulated LOX activity at 12 hpi with the transcript level peaking at −8.92-fold at 72 hpi. Pathogen inoculation alone strongly induced LOX activity at 12 hpi but subsequently caused a sharp decline below baseline levels. The POD transcript was gradually upregulated from 12 to 48 hpi with MeJA and chitosan treatments, followed by a decrease at 72 hpi (Figure 3f, Figure 4f and Figure 5f). The highest expression of this gene reached 10.6-fold in MeJA-treated plants at 48 hpi. Silicon application led to a reverse fluctuation in POD activity, which decreased rapidly after a weak peak of 2.44-fold at 12 hpi. In elicitor-pretreated plants, the pathogen inoculation caused a strong induction of the POD level at 48 hpi. In contrast, the transcript level in plants inoculated with the pathogen alone remained near the baseline level or was downregulated.

## 4. Discussion

The common bean is of substantial agricultural, nutritional, and economic importance worldwide [59]. *Pseudocercospora griseola* constitutes a significant threat to common beans worldwide; however, detailed investigations into more effective control measures apart from chemical methods and resistance sources remained limited [3,5,8]. Elicitors are eco-friendly compounds capable of inducing robust defense mechanisms in plants, thereby offering protection against diseases [13,60]. This study assessed the efficacy of chitosan, methyl jasmonate, and silicon at various concentrations in managing angular leaf spot disease caused by *P*. *griseola* in common beans. The results revealed that elicitor applications at all tested concentrations reduced disease severity, with the most significant reduction observed for silicon, MeJA, and chitosan treatments at 2 mM, 1 mM, and 2 mg/mL concentrations, respectively. Additionally, the enhanced resistance induced by these chemical elicitors was associated with the activation of defense genes in response to pathogen inoculation. The study highlighted the significant effects of these treatments on key signaling molecules involved in regulating plant resistance against pathogens. This research is the first to report the impact of these elicitor applications on defense genes that reduce *P*. *griseola* infection.

Chitosan exhibits significant potential in managing plant pathogenic fungi due to its direct antifungal properties, ability to induce plant defense responses, role in promoting plant health, and environmental safety [14,61]. The effectiveness of chitosan in reducing disease severity across various crops and fungal pathogens is well documented [19,23,25]. However, the efficacy of chitosan treatments on disease resistance could vary depending on factors such as chitosan’s properties, application method, dose, and environmental conditions [14,62]. Our results demonstrated that higher doses of chitosan (low molecular weight, 75–85% deacetylation) obtained from shrimp shells significantly reduced the severity of disease compared to the untreated control and did not cause a phytotoxic effect. The authors of [24] determined that chitosan at a 4% concentration effectively controlled both blast and sheath blight diseases caused by *Pyricularia oryzae* and *Rhizoctonia solani*, respectively. Chitosan at a 0.05% concentration controlled *Colletotrichum* sp. on cucumber [63]. Similarly, chitosan application at a 10 mg/mL concentration reduced and delayed disease symptoms caused by *F*. *circinatum* in *Pinus patula* seedlings [64]. In the current study, a 2% chitosan application proved to be the most effective in controlling *P*. *griseola* infection.

Methyl jasmonate is a potent plant hormone that plays a regulatory role in defense against fungal pathogens [65]. MeJA treatment activates a broad spectrum of defense mechanisms and exhibits antifungal activity depending on its concentration [13,30,66]. Our study indicated that various concentrations of MeJA led to reduced disease development; however, a concentration of 10 mM caused slight phytotoxicity in the plants. Similar findings were reported by [67], who indicated that elevated MeJA doses adversely affected physiological processes, seed germination, seedling emergence, and chlorophyll content in tomato plants. On the other hand, a 0.1 mM MeJA treatment was effective in reducing the disease development caused by *Alternaria porri* f. sp. *solani* in tomato. Pretreatment of Arabidopsis with MeJA led to a significant reduction in disease development by *Alternaria brassicicola*, *Botrytis cinerea*, and *Plectosphaerella cucumerina* [68]. Similarly, MeJA treatment at 0.1 mM inhibited spore germination and mycelial growth of *F*. *oxysporum* f. sp. *lycopersici* in vitro, and reduced disease development in tomato through increased phenylalanine ammonia-lyase activity, aligning with our study’s results [69]. The authors of [70] demonstrated that MeJA has a significant potential for controlling Alternaria leaf spot in tomato by inhibiting fungal growth and spore germination and reducing the incidence and necrotic areas. Similar efficacy has been noted against other pathogens, including *Sclerotinia sclerotiorum*, *Fusarium culmorum*, *Botrytis cinerea*, and *Bipolaris sorokiniana* [32,36,71,72], highlighting MeJA’s potential as an effective and sustainable method for managing fungal diseases in plants.

Silicon has beneficial effects in enhancing resistance to fungal pathogens by promoting the activity of defense-related enzymes and strengthening cell walls [42,73]. The utilization of silicon has been increasingly recognized as an effective approach for managing fungal diseases in plants [73,74]. In our study, root-applied Si significantly reduced the lesion size and number on common bean plants and was notably effective against *P*. *griseola*. These results coincided with the findings of [50], who observed that the foliar applications of potassium silicate at the rates of 0 to 60 g/liter on common plants in field conditions reduced angular leaf spot severity on common beans by 42% and 30% and defoliation by 17% and 33% at pH levels of 5.5 and 10.5, respectively. They also reported a yield increase of 30% and 43% compared to the control at these pH values, respectively. However, they did not address the relationship between silicon treatment and defense-related gene expression in common beans against *P*. *griseola*. These results demonstrated that neither root nor foliar application of silicon to ALS disease in common beans produces significantly different outcomes, independent of other factors. Evaluating the effect of silicon treatment on anthracnose severity caused by *C*. *lindemuthianum* on common beans, Polanco et al. ref. [75] found a 34% reduction in disease severity in common beans grown in a 2 mM silicon solution compared to controls. Similarly, Feng et al. [49] demonstrated the effectiveness of various silicon applications against Sclerotinia stem rot caused by *Sclerotinia sclerotiorum* in rapeseed. The lesion length caused by *Sclerotinia sclerotiorum* on rapeseed stems decreased by 30.5–32.9% with a foliar application of 100 mM Si, while Si application reduced the lesion length on rapeseed stems by 34.9–38.3% when used as basal fertilizer. These results highlighted the broad-spectrum potential of silicon as a viable alternative in integrated disease management strategies.

The activation of PR proteins is a central component of local and systemic acquired resistance responses in plants. These proteins are generally induced in reaction to pathogen infection, elicitor treatments, and environmental stress factors [13,76,77]. The results obtained in this study demonstrated that the expression of PR proteins, including PR1, PR2, and PR3 has a crucial role in resistance response to angular leaf spot disease caused by *P*. *griseola* on common beans. The most significant upregulation was observed in transcript profiles of PR1, PR2, and PR3 proteins following elicitor, elicitor + pathogen, and pathogen applications at 48 hpi. Particularly, *PR2* (β-1,3-glucanase) gene activity exhibited a remarkable increase post-treatments on common bean plants. *PR1* genes, a well-studied group of plant defense genes [78,79], are utilized as molecular markers for the activation of SAR as well as the inhibition of the growth and proliferation of pathogens. They also participate in activating various defense-related genes and pathways [80,81]. Therefore, by evaluating the expression profiles of PR proteins (PR1a, PR1b, PR2, and PR16a and PR16b) on common bean plants after inoculation of *C*. *lindemuthianum* based on host–pathogen interaction alone and without any inducers by qPCR analysis, Borges et al. [82] demonstrated the strong association of PR1 and PR2 proteins with the common bean resistance against pathogens, significantly corroborating our findings. The study examining the transcriptional profile of PR1 proteins in different tissues showed that signal transduction may vary between tissues and showed early recognition in leaves initially, earlier relative expression ratio increase, and then gene activation in epicotyl and hypocotyl tissues [82]. However, some studies have reported that gene expressions in elicitor + pathogen treatments may be suppressed due to synergistic crosstalk between certain signaling pathways [28]. Anisimova et al. [83] reported similar involvement of PR1, PR2, PR4, and PR5 proteins in the resistance of Allium crops to *Fusarium* infections. Our results indicated higher PR protein expression in the early stages following inoculation as compared to uninoculated plants. Similar results regarding the expression profile of PR proteins were also obtained in elicitor applications. Si treatment delayed symptom onset and reduced disease severity in tomato plants infected with *Alternaria solani* by enhancing both the transcript levels of defense signature genes and the activity of antioxidant enzymes [84]. This resistance induction correlated with *PR1*, *PR2*, *PR3*, *LOXD*, and *JERF3* expression and increased activities of antioxidant enzymes SOD, CAT, POD, ascorbate peroxidase (APX), and glutathione reductase (GR). The authors of [23] also observed that chitosan and chitosan nanoparticles mediated resistance to *Fusarium andiyazi*-induced wilt disease in tomato via upregulation of defense-related genes like *PR1*, *β-1,3-glucanase*, *chitinase*, and *PR10* genes.

β-1,3-glucanase and chitinase are well documented for their substantial roles in plant pathogen defense. PR2 proteins (β-1,3-glucanases) strengthen plant resistance by degrading β-1,3-glucans, major components of fungal cell walls [85,86]. PR3 (chitinases) often work in tandem with other PR proteins, such as β-1,3-glucanases, to enhance overall antifungal defense. PR3 also triggers further defense responses, such as activating other PR proteins and producing phytoalexins [76,85,86]. The present study’s results indicated a notable increase in *PR2* (β-1,3-glucanase) gene activity in common bean plants after the applications, emphasizing its pivotal role in disease resistance. The authors of [75] similarly reported increased β-1,3-glucanase activity, with no differences in POX and PPO activities following *C*. *lindemuthianum* infection, while silicon-treated bean plants showed resistance due to the lignification and activities of chitinases, PAL, and LOX. The authors of [71] demonstrated that MeJA or chitosan applications induced β-1,3-glucanases and chitinases, reducing *Botrytis cinerea*-induced gray mold decay in strawberries. Similarly, MeJA, Si, and chitosan treatments increased disease resistance across various plant species by inducing PR proteins, including PR1, PR2, and PR3 [30,61,74]. These findings underscored the significant role of the elicitors in the activation of *PR* genes.

The expression of enzymes associated with defense mechanisms is essential for host resistance to pathogenic attacks. Phenylalanine ammonia-lyase (*PAL*) genes contribute to plant defense against fungal pathogens by facilitating the biosynthesis of phytoalexins, lignin, salicylic acid, and various phenolic compounds [61]. Our results indicated that PAL activity peaked in common bean plants treated with MeJA, chitosan, and silicon at 48 hpi, and this enhanced PAL activity correlated with increased resistance to *P*. *griseola*. This result was in line with [87], who observed a significant rise in the expression of *PAL* and *LOX* genes in perennial ryegrass grown in Si-amended soil and inoculated with *Magnaporthe oryzae*. Similarly, chitosan treatment against green mold in citrus fruit induced the activities of POX and PAL in infected tissues [88]. The authors of [32] indicated that MeJA treatment in wheat plants following *F*. *culmorum* inoculation strongly induced the expression of PAL and LOX transcripts. These findings suggested that chemical elicitors were significantly involved in the regulation of PAL enzyme activity in plants.

Peroxidase (POD) enzymes are involved in oxidative burst, lignin biosynthesis, and other defense-related processes. Upregulation of *POD* genes is frequently associated with enhanced resistance to fungal pathogens. In this study, MeJA and chitosan treatments upregulated POD activity until 48 hpi, while Si treatment caused an early-stage expression enhancement, followed by a decrease. Similarly, the application of 1.5 mM MeJA in banana plants reduced disease incidence and severity caused by *Fusarium oxysporum*, accompanied by higher activities of SOD, POD, PPO, CAT, and PAL [89]. Root-applied Si treatment significantly induced the activities of chitinase, POD, and PPO in cucumber inoculated with *Podosphaera xanthii* [90]. The enhancement of peroxidase and polyphenol oxidase expression following the injection of chitosan at 1 mg/mL into roots contributed to increased resistance in date palm against *Fusarium oxysporum* f. sp. *albedinis* [91]. In this study, *LOX* gene expression initially increased in the early stages following all treatments but subsequently decreased over time. The results indicated that LOX activity made a limited contribution to controlling ALS disease in common beans. This finding was consistent with the results of [92], who observed that chitosan treatment upregulated *CHI* and *PAL* genes but did not significantly induce *LOX* genes in resistance to stem-end rot and anthracnose diseases in avocados. Similarly, chemical elicitor treatments have been reported to enhance resistance in a wide range of host species such as peach, grape, soybean, rice, and citrus by increasing the activities of defense-related enzymes in response to pathogen infections [93,94,95,96,97].

## 5. Conclusions

In summary, this study revealed that the application of chitosan, methyl jasmonate, and silicon led to a substantial reduction in the development of angular leaf spot in common beans. These findings underscored the broad-spectrum potential of chemical elicitors as crucial elements in integrated disease management strategies. Additionally, the study assessed the potential involvement of PR-proteins and the defense enzymes of common beans in response to *P*. *griseola*. Enhanced resistance to angular leaf spot in common beans was associated with the expression of *PR1*, *PR2*, *PR3*, *PAL*, *POD*, and *LOX* genes. Furthermore, the findings offered valuable insights into the kinetics of the defense response of common beans against this pathogen. However, to improve the efficacy of chemical elicitors in plant disease management strategies, further comprehensive studies are needed on application methods, formulation strategies, dosing, and combined treatments with biocontrol agents and fungicides of chemical elicitors as well as the defense pathways and mechanisms underlying host resistance.

## Figures and Tables

**Table 1:** List of primer sequences used for qPCR.

Defense-Related Genes/Enzymes	Forward Primer (5′–3′)	Reverse Primer (5′–3′)
Peroxidase (POD)	TCC TTT TCA GCA CTT TCA CT	AGA AAG CAG TGT TCT TGT GG
Pathogenesis-related 1 (*PR1*)	AAA GCC AAG AGC GAT TCT CTT TTCA	GAA CAC TCT GAT TTG ATA ACA CTTC
β-1,3 endoglucanase (*PR2*)	GAA GAT GAG CTC AAA GCT GGT AA	CAA GGA TTG GCC AAA AGG TA
Chitinase class I (*PR3*)	ATT GTT GTG CCA ATC CCT TT	CAC CGC CAT ACA GTT CAA AA
Lipoxygenase (LOX)	AGC ACT GTG CCT GTT TTC AGT	AAC ACA CGA GAA GAT TCA ACCA
Phenylalanine ammonia-lyase (PAL)	GAC ACA CAA GTT GAA GCA CCA	TGC AGC TTC TTA GCA TCC TTC
*Actin*	TGC ATA CGT TGG TGA TGA GG	AGC CTT GGG GTT AAG AGG AG

## Data Availability

The original contributions presented in this study are included in the article; further inquiries can be directed to the corresponding author.
